# Structural biology meets typography: using protein structures to inspire creative expression and connect diverse audiences

**DOI:** 10.3389/fbinf.2025.1589122

**Published:** 2025-05-08

**Authors:** Leonora Martínez-Núñez

**Affiliations:** Department of Biochemistry and Molecular Biotechnology, University of Massachusetts Chan Medical School, Worcester, MA, United States

**Keywords:** protein structure, molecular visualization, scientific outreach, structural biology, 3D modelling, molecular data, typography, creative expression

## Abstract

Proteins are complex molecular machines with specific structures that determine their function. Advances in structural bioinformatics and visualization have expanded access to molecular data, most notably through the Protein Data Bank (PDB). This perspective explores the intersection between structural biology and typography, integrating a protein alphabet with the 36 Days of Type design project. Using ChimeraX, Blender and Molecular Nodes, 3D molecular models were processed, stylized, and shared on social media under the #36daysoftype hashtag, which led to engagement across a diverse audience. This work was also presented at VIZBI 2024 conference and influenced the VIZBI 2025 conference logo design. This project frames the role of scientific illustration and visual arts in connecting disciplines, boosting public engagement, and encouraging interdisciplinary collaboration, while also inspiring future applications like biology-inspired typography to enhance scientific literacy.

## 1 Introduction

Nearly every function in living organisms, from breathing to muscle growth and digestion relies on proteins, the nanoscopic molecular machines that are essential for life. Proteins contain smaller units known as amino acids, assembled and linked like beads on a string by peptide bonds. The linear sequence of amino acids is the primary structure of a protein. Proteins acquire their secondary structure based on the chemical properties of their amino acids which fold into patterns such as alpha-helices and beta-sheets due to the formation of hydrogen bonds. The alpha-helices and beta-sheets can then fold into a tertiary structure with a three-dimensional shape that is stabilized by chemical and physical interactions. Multiple proteins can assemble into large complexes with collaborative functions, giving them a quaternary structure (Stollar and Smith, 2020). Correct protein folding is essential for overall function, because small errors or misfolds can completely abolish protein functionality, which is determined by sequence, structure and ultimately, interactions with other molecules (Dobson, 2003). Loss of protein function due to misfolding is associated with several human diseases, including Alzheimer’s disease, Parkinson’s disease, amyotrophic lateral sclerosis (ALS), cystic fibrosis, type 2 diabetes, among others (Soto and Pritzkow, 2018; Mukherjee et al., 2015; Yadav et al., 2019).

Proteins simultaneously present as complex machines and works of art shaped by evolution and physics, with intricate patterns invisible to the naked eye and often observed by a niche group of specialist researchers. However, thanks to techniques such as X-ray Crystallography, Cryo-Electron Microscopy (Cryo-EM), and the development of computational biology methods like protein structure predictions based on artificial intelligence (AI) algorithms, combined with structural bioinformatics and visualization applications, “observing” proteins in three-dimensional space has become a reality (Yip et al., 2020; García-Nafría and Tate, 2021; Jumper et al., 2021). Everyone can access the latest structural data through the Protein Data Bank (PDB), which primarily stores proteins as well as DNA and RNA data (Berman et al., 2000). The PDB contains multiple educational resources, particularly the “Molecule of the Month”, created by Dr. David Godsell as a series of short pieces about selected molecules from the PDB, their structure, function, and importance in biomedicine and in daily life (Goodsell et al., 2020, https://pdb101.rcsb.org/motm/motm-about).

The above-mentioned resources are open source available to everyone, but the reality is that only a small group of people with specialized interest in protein biology utilize these datasets and take advantage of the scientific information they provide.

In this perspective article I will highlight my work using protein structures from the PDB in a typography experiment, which was inspired by the Protein Alphabet originally curated by Mark Howard (Howarth, 2015). My approach uses structural and computational biology tools, novel molecular visualization methods combined with the typographic social media trend 36 Days of Type as a new opportunity for scientific outreach through design and artistic expression.

## 2 The convergence of protein structures and typography

Typography and protein structures may appear as unrelated fields, yet they converge at one point: encoding complex information within constructed forms. They both rely on encoded information to create meaning and function. Just as alphabets form words to convey ideas and meaning, the genetic material in our cells, DNA, arranges four nucleotides—A, T, C, and G—into a code for building proteins. The triplet codon system, where three nucleotides specify an amino acid, echoes how letters form words.

Protein structures translate to biological functions within precise molecular architectures as typography transforms language into visual symbols with meaning and style. By applying principles of type design to protein visualization, we can explore new ways of presenting and communicating biological data, inform different ways to teach molecular biology, and generate novel biology inspired art.

For the work dissected in this perspective, I utilized a previously curated English alphabet of protein structures (Howarth, 2015). Howarth’s alphabet is an outreach activity that explores the diversity and importance of proteins and provides customized, rainbow-colour visuals of names using the protein structures (https://www.nigms.nih.gov/education/Pages/Protein-Alphabet.aspx).

I discovered the protein alphabet while exploring outreach activities during my role as Sr program manager for outreach at Umass Chan Medical School. During this time, my career focus shifted from the research lab towards scientific illustration, design and communication. At the time, I have been following the 36 Days of Type project. Mainly exhibited on social media, the project invites designers, illustrators, and visual artists to express their unique interpretation of the letters and numbers of the Latin alphabet for 36 days (Tchaco, 2020). The result is a spectacular display of the same symbols visualized through thousands of different perspectives.

## 3 The protein alphabet meets 36 days of type

I decided to create a collection of tailored illustrations using PDBs from the protein alphabet to share one new design on social media each day of the 36-day type challenge.

I used the PDBs listed in Howarth’s protein alphabet, but exchanged the letter I for the PDB 3B5N, a structure of the yeast plasma membrane SNARE complex relevant to my postdoctoral research.

I followed two different methods to obtain the molecular 3D models. For the first approach, the PDB coordinates were downloaded into the molecular visualization software ChimeraX (Pettersen et al., 2021, https://www.cgl.ucsf.edu/chimerax/). Once in ChimeraX, I experimented with different representations of atoms and secondary structures, focusing on interesting domains within the protein or highlighting ligands and co-factors. After the molecular representation was finalized, the 3D model was exported in GLTF format (.glb file, https://www.rbvi.ucsf.edu/chimerax/data/texture-may2021/export_3d.html) and then imported into the 3D software Blender V. 4.2 (https://www.blender.org). The second approach to obtain a molecular model leverages the molecular nodes add-on created by Brady Johnston (Johnston, 2024). This add-on code, openly available on github (https://github.com/BradyAJohnston/MolecularNodes) enables quick import and visualisation of structural biology data inside of Blender and allows the 3D program to understand the molecular data. In light of creative freedom, the letters B, D, E, I, J, L, M and N were processed using ChimeraX and A, C, F, G, H, K O using the molecular nodes add-on. The letters and corresponding PDB numbers, as well as the post-processing software are listed in Supplementary Table S1.

Once the 3D models were in Blender, a complete scene for each alphabet letter was created. The molecular models were strategically placed within the scene to show the best conformation that resembled the letter of the alphabet. The 3D models were then stylized by adding textures, and colours. Finally, the complete illustrations were rendered using the Cycles engine.

Fifteen illustrations were crafted (https://www.leonoramartinez.com/musings-of-the-protein-alphabet) and posted on social media under the hashtag #36daysoftype to participate in the challenge (https://www.36daysoftype.com/). Although I did not post for 36 consecutive days, creating these illustrations allowed me to showcase the beauty of protein structures to diverse audiences, connecting typography design, illustration, visual arts and structural biology.

The reactions to the posts on social media were interesting. According to the metrics provided by the platforms (e.g., Instagram and X), some of the posts reached up to 649 external accounts (Instagram: *unique users who have seen your post at least once*) while some posts got up to 6,447 impressions (X: *the total number of times the content has been viewed. Metrics for every post are listed in the* Supplementary Table S1). A broad pool of users, including scientists, designers, artists, and daily social media users, engaged with the visual content created during the experiment.

## 4 VIZBI entry for Art & Biology 2024

**FIGURE 1:**
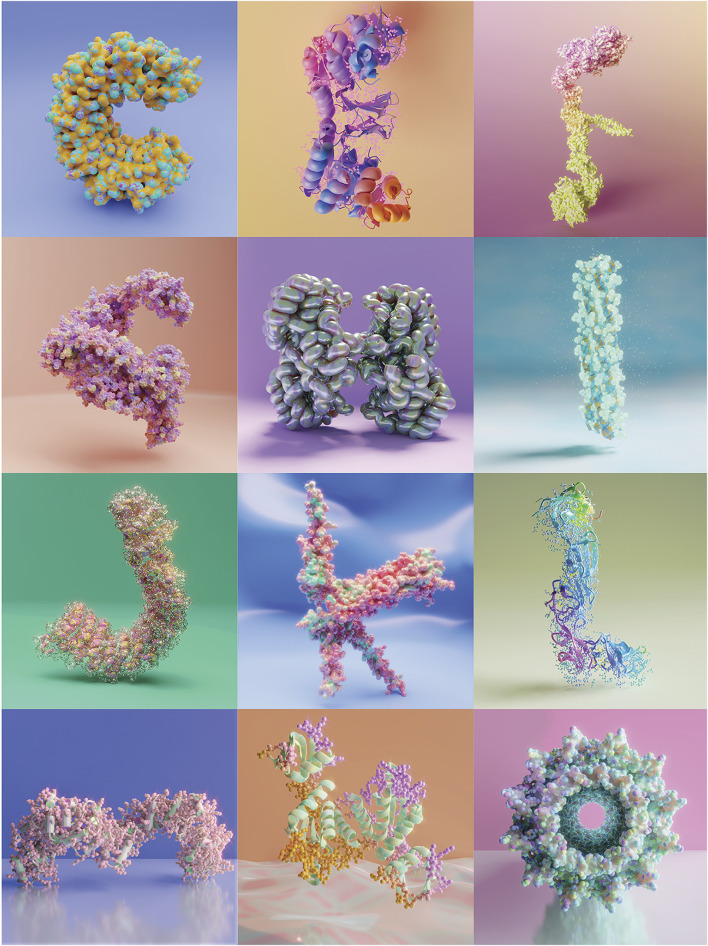
Artwork presented at the VIZBI 2024 Conference, “Musings on the protein alphabet”. Awarded best poster in the Art & Biology category.

The annual Visualizing Biological Data (VIZBI) international conference brings together researchers developing and using computational visualization to address questions in diverse biological fields; it additionally attracts medical illustrators, graphic designers, and graphic artists, thus embodying a true cross-disciplinary community. The conference invites participants to submit posters, lightning talks, and biology-inspired artworks. To participate in the Art & Biology category, I submitted a poster featuring twelve of the fifteen illustrations I created for the 36 Days of Type challenge. Arranged in a three-by-four grid, the artwork “Musings on the Protein Alphabet”, deviated from the conventional typography approach by incorporating a biological element—crystal protein structures sourced from the PDB (Figure 1). The artwork was awarded the best poster in the Art and Biology category during the VIZBI Conference 2024.

## 5 Expanding the biology-inspired type: Logo for VIZBI 2025

The VIZBI community has been invaluable to me. Over the past few years, it has provided the opportunity to connect with incredible scientists and creatives during the annual the conference. I was thankful for the opportunity to participate creatively in the concept for the 2025 conference logo, which aimed to expand the data-driven typography from proteins to a broader biology-inspired type relevant to the extensive range of topics discussed during the conference.

**FIGURE 2:**
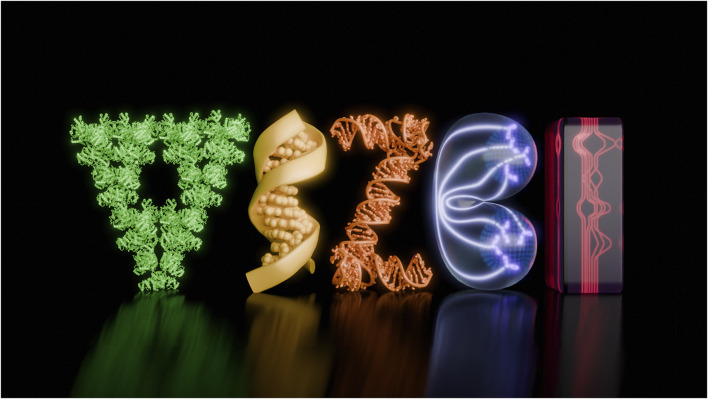
Logo for the VIZBI 2025 Conference.

My design of the VIZBI logo for 2025 (Figure 2; Supplementary Video S1) is a composition of the following models from left to right: the letter “V” depicts one segment (18mer, level 1) of the Sierpiński triangle fractals formed by the self-assembly of the citrate synthase from *S. elongatus* (Sendker et al., 2024. PDB 8RJK). The first letter “I,” represents the crystal structure of the synthetic DNA dodecamer, reported in 1981 (Drew et al., 1981). This structure, annotated as PDB 1BNA, provides insights into the fine-scale geometry of B-DNA, the predominant form of DNA in cells. The letter “Z” depicts the crystal structure of an adenovirus virus-associated RNA (PDB 6OL3), which was the first high-resolution structure of a noncoding virus-associated RNA produced in high amounts by adenoviruses to evade host antiviral defences (Hood et al., 2019). For the VIZBI logo, 1.5 copies of the original structure were used and manually compiled to resemble the letter “Z.” The letter “B” is represented by a 3D-generated model of a cell undergoing division (Scholey et al., 2003). It illustrates a layout of cytoskeletal components, chromosomes and nuclei surrounded by a plasma membrane just before cytokinesis. The final letter “I,” is a creative representation of a sequence tube map with genome assembly data inspired by the graphs from the human pangenome project (Liao et al., 2023).

The molecular PDB coordinates were processed in ChimeraX, then exported the 3D models in GLTF format for use in Blender. Additional models were then manually created in Blender, where compositions were generated and incorporated into a scene to create the logo illustration. Blender V. 4.2 was used to render the illustration and animation and Adobe Photoshop was used for post-production of the animated version. The resulting visuals are shown on the main page of the VIZBI 2025 conference (https://vizbi.org/2025/).

## 6 Discussion

While proteins are widely recognized for their role in nutrition, health, and fitness, their remarkable molecular structures remain largely unfamiliar to those outside specialized scientific fields. By implementing and exploring methods discussed in this perspective including merging structural biology with illustration and design, while also utilizing scientific knowledge across fields, this work demonstrates how we as scientists can make lesser-known scientific concepts more accessible.

For this work, the PDB database (https://www.rcsb.org/) was leveraged and most of the protein alphabet curated by Mark Howarth (Howarth, 2015) was used. The PDB stores 232,059 structures and 1,068,577 computer-predicted structures to date, so accessibility to interesting-looking structures is not straightforward; thus, the protein alphabet provided a valuable data source and starting point. I decided to exchange the letter “I” suggested for a protein structure relevant to my postdoctoral work that I was familiar with. This seemingly small change suggests that additional protein structures might work as typographic symbols and could be incorporated into a more extensive database for use in outreach and education.

The existence of molecular visualization tools such as ChimeraX facilitates the creation of illustrations for scientific research communications, but it can also be creatively limiting. Scientists are always looking for new ways of improving the visuals to showcase their work. Combining molecular visualization software with 3D programs such as Blender provides a new way to do this. Blender is an open-source 3D computer graphics software used to create award-winning animated films, visual effects, art, motion graphics, virtual reality and video games (Blender Online Community, 2024). However, it does not inherently process molecular data. The Molecular Nodes add-on (Johnston, 2024) has provided a novel tool which has revolutionized how structural biologists visualize and share their work with the community. While mastering these tools presents a steep learning curve, numerous online resources are available to support users.

The graphic design trend 36 Days of Type on social media provided a great platform for this cross-disciplinary work. Participating in the project gave me the canvas to be bold with the environment design and the final illustrations, which are depicted in a way that does not conform to scientific accuracy beyond the molecular structure of the protein. Each protein illustration resembles a parallel world. I experimented with different light sources, backgrounds, textures, and surface materials like candy, glass, glossy, slime and iridescent metal. The goal was to create an eye-catching visual with an artistic representation of scientific data, following design principles and maintaining a personal aesthetic.

The metrics collected from the social media platforms showed modest engagement. Changing algorithms, number of followers, and the use of scientific captions are the most likely limiting factors when using social media as a tool for scientific outreach. Combining eye-catching visuals with a caption crafted for a general audience would be factors recommended for success in the future. Although it is unclear whether the 36 Days of Challenge social media project will continue, as it is currently in hiatus, further participation would be welcomed and personally pursued. Perhaps more scientific communicators, and scientists, may begin to adopt it and incorporate it into other social media projects such as #sciartseptember or #inkoctober.

The cross-disciplinary work reviewed here was recognized at the VIZBI 2024 conference and provided me with the opportunity to create the VIZBI 2025 animated logo. The design process involved selecting molecular structures or creating new 3D models visually representing the VIZBI conference core themes—DNA to ecosystems—while ensuring the final logo was attractive and meaningful. This process highlights how scientific visualization can be used in branding and identity design to reinforce a conference’s mission and engage participants through an instantly recognizable visual language. The VIZBI 2025 logo is the preliminary work to further build a biology-inspired type that can be used in science communication and education.

Animations, interactive displays, virtual reality, and even physical sculptures and ceramics can help emphasize the art and design inherent in biology. Moreover, collaborating with typographers and graphic designers could further refine and expand this idea within mainstream design culture, with the aim of increasing science literacy within the broader community.

## Data Availability

The original contributions presented in the study are included in the article/Supplementary Material, further inquiries can be directed to the corresponding author.
