# Supplementary material for: Structural biology meets typography: using protein structures to inspire creative expression and connect diverse audiences
Source: Front Bioinform. 2025 May 8;5:1589122. doi: 10.3389/fbinf.2025.1589122 (PMC12094914; doi:10.3389/fbinf.2025.1589122)
Supplement: Supplementary file 1 [file Table1.docx]

**Supplementary table 1. Summary of letters of the alphabet, PDB numbers and processing software used for project 36 Days of type including social media metrics of each letter posted on Instagram and X.**

| Letter | PDB number | ChimeraX + Blender | Molecular Nodes + Blender |  | Social media metrics | | | Curated by |
| --- | --- | --- | --- | --- | --- | --- | --- | --- |
|  |  |  |  | Instagram accounts reached | Instagram  impressions | X engagement | X impressions |  |
| A | [3IFZ](https://www.rcsb.org/structure/3IFZ) |  | 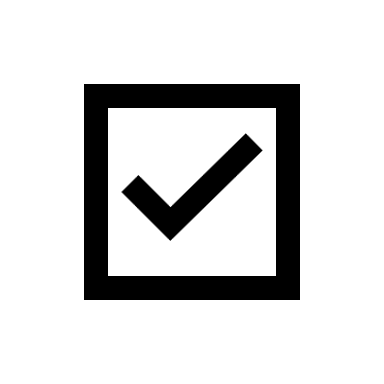 | 330 | 375 | 94 | 2,628 | Howard, M. 2015 |
| B | [2QYC](https://www.rcsb.org/structure/2QYC) | 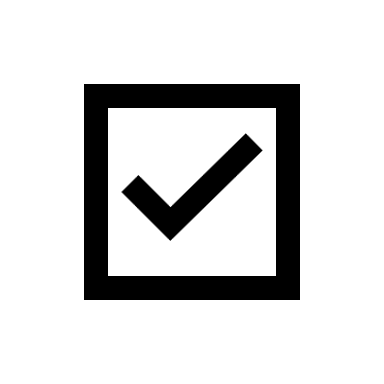 |  | 236 | 260 | 33 | 593 | Howard, M. 2015 |
| C | [2BNH](https://www.rcsb.org/structure/2BNH) |  | 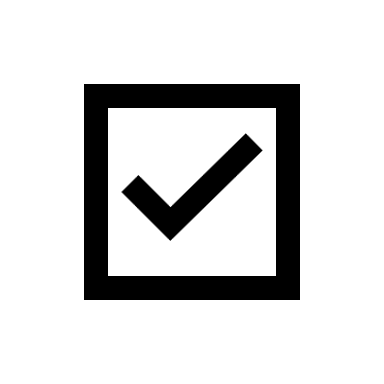 | 649 | 738 | 163 | 2,272 | Howard, M. 2015 |
| D | [4J3O](https://www.rcsb.org/structure/4J3O) | 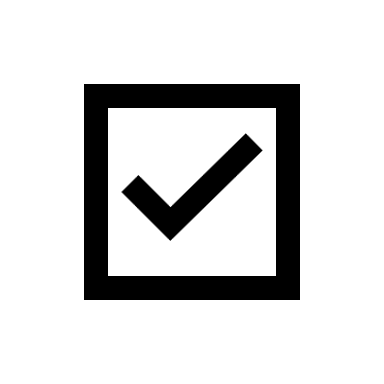 |  | 273 | 316 | 37 | 634 | Howard, M. 2015 |
| E | [2Q5R](https://www.rcsb.org/structure/2Q5R) | 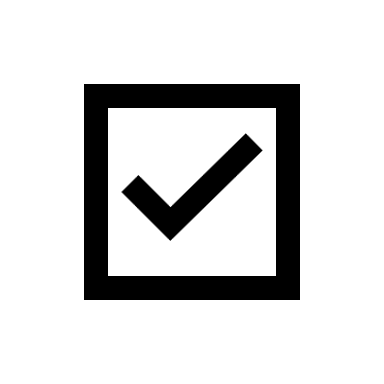 |  | 232 | 292 | 225 | 3,138 | Howard, M. 2015 |
| F | [3J04](https://www.rcsb.org/structure/3J04) |  | 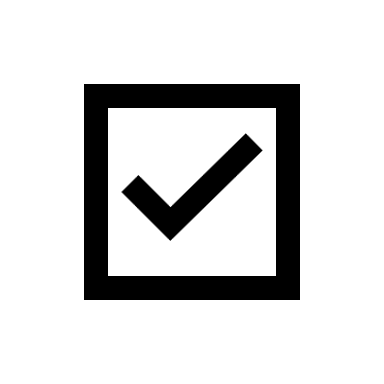 | 334 | 399 | 135 | 1,976 | Howard, M. 2015 |
| G | [4U48](https://www.rcsb.org/structure/4U48) |  | 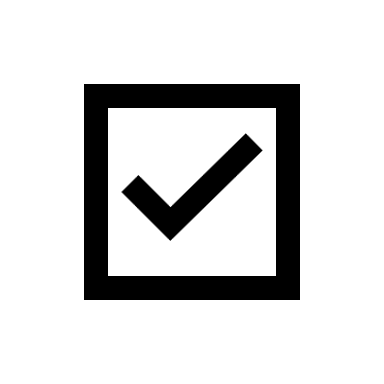 | 254 | 284 | 153 | 2,940 | Howard, M. 2015 |
| H | [1XU9](https://www.rcsb.org/structure/1XU9) |  | 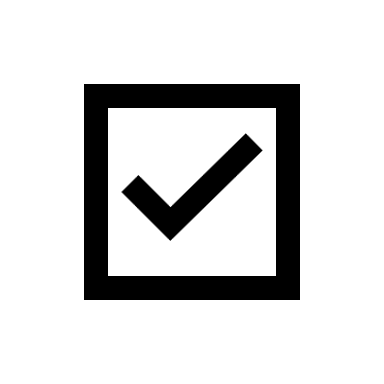 | 235 | 264 | 101 | 1,865 | Howard, M. 2015 |
| I | [3B5N](https://www.rcsb.org/structure/3B5N) | 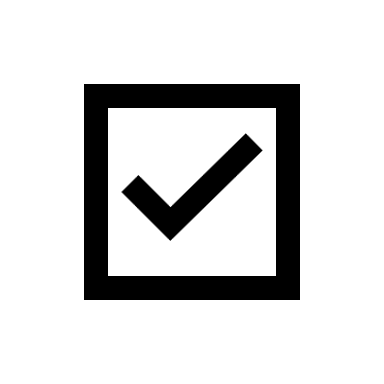 |  | 252 | 279 | 81 | 1,417 | Martínez-Núñez, L. 2024 |
| J | [1B3U](https://www.rcsb.org/structure/1B3U) | 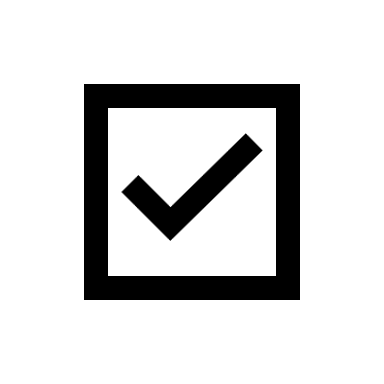 |  | 207 | 245 | 106 | 1,411 | Howard, M. 2015 |
| K | [4OX0](https://www.rcsb.org/structure/4OX0) |  | 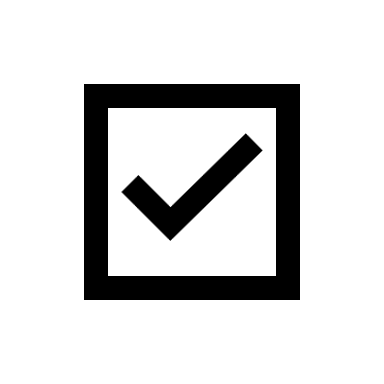 | 309 | 333 | 51 | 1,312 | Howard, M. 2015 |
| L | [1UEB](https://www.rcsb.org/structure/1UEB) | 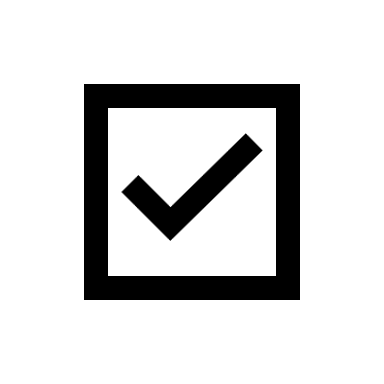 |  | 285 | 322 | 132 | 2,620 | Howard, M. 2015 |
| M | [1OU5](https://www.rcsb.org/structure/1OU5) | 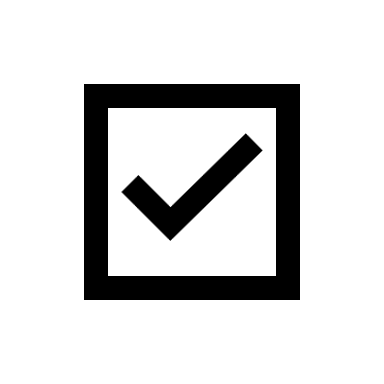 |  | 291 | 333 | 94 | 1,488 | Howard, M. 2015 |
| N | [1Z85](https://www.rcsb.org/structure/1Z85) | 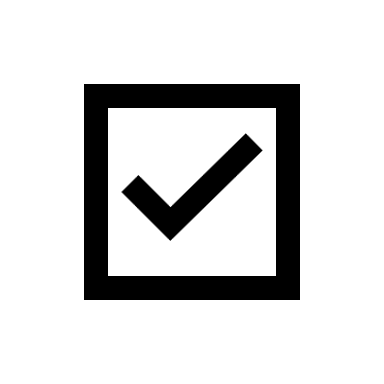 |  | 291 | 345 | 80 | 1,141 | Howard, M. 2015 |
| O | [2WCD](https://www.rcsb.org/structure/2WCD) |  | 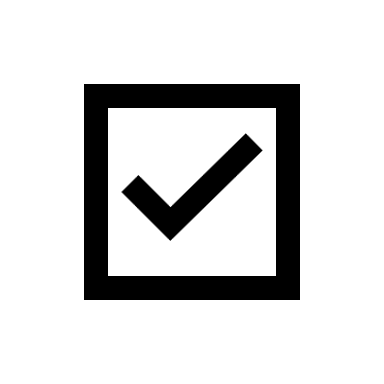 | 365 | 475 | 510 | 6,447 | Howard, M. 2015 |
